# The Aging Kidney and Exercise Training Study: protocol for a randomized controlled trial within cohorts (TwiCs) study

**DOI:** 10.1186/s12882-025-04471-y

**Published:** 2025-09-26

**Authors:** Keisei Kosaki, Shoya Mori, Hayate Namatame, Riri Kobayashi, Shun Yoshikoshi, Takashi Tarumi, Toshiaki Usui, Chie Saito, Masahiko Gosho, Yoshio Nakata, Seiji Maeda, Makoto Kuro-o, Kunihiro Yamagata

**Affiliations:** 1 Institute of Health and Sport Sciences, University of Tsukuba, 1-1-1 Tennodai, Tsukuba, Ibaraki, 305-8574 Japan; 2 Advanced Research Initiative for Human High Performance, University of Tsukuba, Ibaraki, Japan; 3 Division of Preventive and Sports Nephrology, Graduate School of Comprehensive Human Sciences, University of Tsukuba, Ibaraki, Japan; 4 Department of Nutritional Science, Faculty of Applied Biosciences, Tokyo University of Agriculture, Tokyo, Japan; 5 Department of Cardiology, Institute of Medicine, University of Tsukuba, Ibaraki, Japan; 6 Graduate School of Comprehensive Human Sciences, University of Tsukuba, Ibaraki, Japan; 7 Integrated Research Center for Self–Care Technology, National Institute of Advanced Industrial Science and Technology, Ibaraki, Japan; 8 Department of Nephrology, Institute of Medicine, University of Tsukuba, Ibaraki, Japan; 9 Department of Biostatistics, Institute of Medicine, University of Tsukuba, Ibaraki, Japan; 10 Faculty of Sport Sciences, Waseda University, Saitama, Japan; 11 Division of Mineral Metabolism, Center for Molecular Medicine, Jichi Medical University, Tochigi, Japan

**Keywords:** Multicomponent training, Kidney function decline, Renal hemodynamics, Mineral metabolism, Cardiovascular function, Sports nephrology

## Abstract

**Introduction:**

Chronic kidney disease (CKD) is a growing global public health concern, particularly in aging population. Despite the strong epidemiological evidence linking physical activity to improved kidney outcomes, interventional findings remain scarce and inconsistent. The primary purpose of the Aging Kidney and Exercise Training Study (AKETS) is to investigate whether a structured exercise program would attenuate the kidney function decline in middle–aged and older adults using a trial within cohorts (TwiCs) design.

**Methods:**

The AKETS is a 24–month, community–based, randomized controlled trial embedded in the prospective Aging Kidney Study cohort, which started in November 2018. A total of 141 participants aged 40–90 years are enrolled and allocated to one of the following three groups: (1) exercise intervention, (2) stretching intervention (active control), and (3) usual lifestyle (time control). The exercise program comprises individualized multicomponent training, including aerobic, resistance, and balance exercises, performed three times per week over 12 months.

**Results:**

The primary outcome is the slope of the estimated glomerular filtration rate (eGFR) based on serum cystatin C. The secondary outcomes include renal hemodynamics, mineral metabolism, cardiovascular function, body composition, muscle strength, and physical performance. At baseline, the exercise, stretching, and usual lifestyle groups were well matched for key demographic and clinical characteristics, such as age, proportion of women, and eGFR.

**Conclusion:**

Findings from the AKETS will allow for a robust evaluation of exercise as a primary preventive strategy for CKD and are expected to inform future nonpharmacological interventions targeting kidney health in the aging population.

**Trial registration:**

This study is registered at the University Hospital Medical Information Network Clinical Trials Registry as UMIN000055825. Registered on the 13th of October 2024.

**Supplementary Information:**

The online version contains supplementary material available at 10.1186/s12882-025-04471-y.

## Introduction

Chronic kidney disease (CKD), defined by persistent abnormality of kidney structure and/or function, is a major global public health concern [1, 2]. The prevalence of CKD is expected to increase rapidly as the average age of the world’s population continues to rise [3]. In fact, in Japan, which has entered an ultra–aged society, approximately 15 million people (or one in seven adults) have developed CKD [4]. CKD often develops without noticeable symptoms [5], leading to multiple adverse outcomes, including cardiovascular events and renal replacement therapy (dialysis or kidney transplantation), cognitive and physical decline, hospitalization, and all–cause mortality [6, 7]. Although advances in pharmacotherapy [8, 9] —such as renin–angiotensin system inhibitors and sodium–glucose cotransporter 2 inhibitors— have improved control of established risk factors (i.e., hypertension and diabetes), the CKD population continues to increase. This highlights the persistent lack of effective non–pharmacological strategies to slow kidney function decline, particularly among middle–aged and older adults.

Structural and functional changes in the kidneys that occur as part of the aging process constitute a fundamental pathophysiological basis for CKD and are crucial factors for determining the subsequent severity of CKD progression [10, 11]. Moreover, renal hemodynamic alterations [12] and disturbed mineral metabolism [13] observed from early midlife may contribute to the pathogenesis of CKD. Therefore, suppressing or delaying these changes may effectively mitigate CKD progression in later life. Notably, a decline in the glomerular filtration rate beyond physiological tolerance limits (i.e., rapid kidney function decline), facilitated by deterioration in renal hemodynamics and mineral metabolism, is closely linked to long–term renal outcomes, making it a primary target for preventive interventions.

Several cross–sectional studies have reported that individuals with higher levels of physical activity and performance tend to have better kidney function [14–18]. Furthermore, longitudinal studies have demonstrated an independent association of physical activity and fitness with preventing CKD development and rapid kidney function decline [19–23]. Recent evidence from animal model studies also indicates the potential renoprotective effects of long–term exercise training in various CKD models [24–28]. These epidemiological and basic findings suggest that exercise training aimed at improving physical activity and fitness could be an effective strategy for preventing CKD development [29, 30]. However, it remains possible that declines in kidney function may lead to reduced muscle mass and impaired physical performance, subsequently resulting in lower physical activity levels (i.e., reverse causation). Indeed, evidence in humans from the limited number of randomized controlled trials (RCTs) investigating whether exercise training benefits kidney health has been inconsistent [31–34]. Therefore, the renoprotective effects of exercise training remain uncertain and controversial, highlighting the need for rigorously designed interventional studies to clarify causality.

The inconsistent findings from exercise–nephrology RCTs may stem from the possibility that kidney aging is irreversible and progresses slowly with significant individual variations. Thus, when investigating the effects of exercise training on kidney health, within–group (intra–individual) comparisons may be more critical than between–group (inter–individual) comparisons. To achieve both comparisons, the trial within cohorts (TwiCs) study design, in which the intervention study is performed within a prospective observational cohort with regular measurements [35, 36], is considered more appropriate than conventional standard RCTs. The TwiCs study design allows for an evaluation of not only within–group changes and between–group differences but also the long–term effects of exercise training intervention, because regular measurements in the prospective cohort will be continued after the completion of the intervention period [37].

By using the TwiCs study design, the current study involving middle–aged and older adults aims to investigate whether a 1–year progressive moderate– to high–intensity exercise training program can slow kidney function decline and renal hemodynamics/mineral metabolism deteriorations. The hypotheses for this study are as follows: (1) 1–year of structured exercise training will attenuate the kidney function decline while improving renal hemodynamics and mineral metabolic status. Specifically, we hypothesize that the annualized rate of kidney function decline during the 1–year intervention period will be smaller than the annualized rate observed during the 5–year pre–intervention observational period within the same individuals assigned to the exercise group. (2) Additionally, we hypothesize that increases in physical activity and fitness levels will be positively associated with favorable changes in kidney function. To our knowledge, this study is the first to apply the TwiCs study design in the field of sports nephrology. The current study is expected to provide new insights regarding the effectiveness of nonpharmaceutical interventions for CKD development.

## Methods

### Study design, procedure, and ethics

**Fig. 1:**
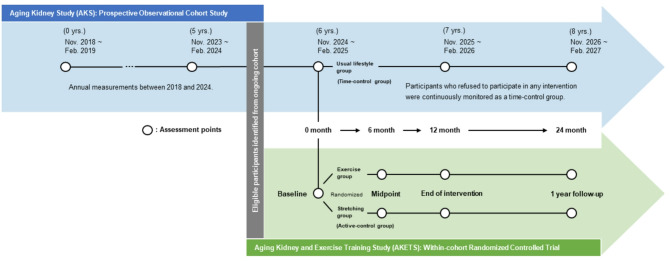
Study overview. The Aging Kidney and Exercise Training Study (AKETS) is a 24–month, community–based, two–arm RCT that employs the trial within cohorts (TwiCs) study design. The AKETS protocol includes four assessment time points: baseline, 6 months (midpoint), 12 months (end of intervention), and 24 months (follow–up)

The Aging Kidney and Exercise Training Study (AKETS) is a 24–month, community–based, two–arm RCT using the TwiCs study design (Fig. 1) and is conducted within the Aging Kidney Study (AKS) cohort, which started in November 2018 with the aim of investigating the associations between age–related changes in mineral metabolism and kidney health in individuals with and without CKD (UMIN000034741). The AKS cohort is a uniquely comprehensive dataset encompassing physiological, biochemical, behavioral, and physical performance measures relevant to kidney health, enabling multifactorial analyses of potential risk and protective factors associated with age–related kidney function decline. Cross–sectional analyses from the AKS cohort have already identified some novel lifestyle factors of kidney health [38–43], highlighting the potential importance of non–pharmacological strategies to slow kidney function decline. Such preliminary evidence from the AKS cohort provides a strong rationale for conducting the current study. The AKETS protocol includes four assessment time points: baseline, 6 months (midpoint), 12 months (end of intervention), and 24 months (follow–up) (Fig. 1).

Ethical approval was granted by the Institutional Review Board of the University of Tsukuba (approval no. 2029) in accordance with the guidelines of the Declaration of Helsinki and Belmont Report. Written informed consent has been obtained from all participants before participating in the study. This study is registered at the University Hospital Medical Information Network Clinical Trials Registry as UMIN000055825 and is funded by the Japan Agency for Medical Research and Development, Core Research for Evolutional Science and Technology, Cross–Ministerial Strategic Innovation Promotion Program, and Japan Society for the Promotion of Science.

### Participants

Middle–aged and older adults deemed eligible to participate in the intervention groups of AKETS were enrolled from the AKS cohort if they satisfied the following inclusion criteria: (1) aged 40–90 years; (2) estimated glomerular filtration rate (eGFR) calculated based on serum cystatin C levels > 30 mL/min/1.73 m^2^; (3) no restrictions on exercise imposed by a medical doctor; (4) no current severe illnesses or significant past medical history; (5) agreement to the terms and conditions of the medical fitness center; and (6) absence of issues that would affect participation in the study. Participants with contraindications (e.g., neurological and walking problems) to exercise training are excluded. Individuals interested in participating are provided with written and oral information from the researchers regarding the study aims, procedures, confidentiality of personal information, possible benefits and risks, and coping strategies. Only participants who provide written informed consent were included in the current trial. A total of 203 middle–aged and older adults who met the eligibility criteria received invitation letters and emails. Of them, 141 participated in the baseline assessment, whereas the remaining 62 declined to participate for personal reasons such as work commitments, lack of time, and living too far away to regularly attend the fitness center.

### Randomization and blinding

**Fig. 2:**
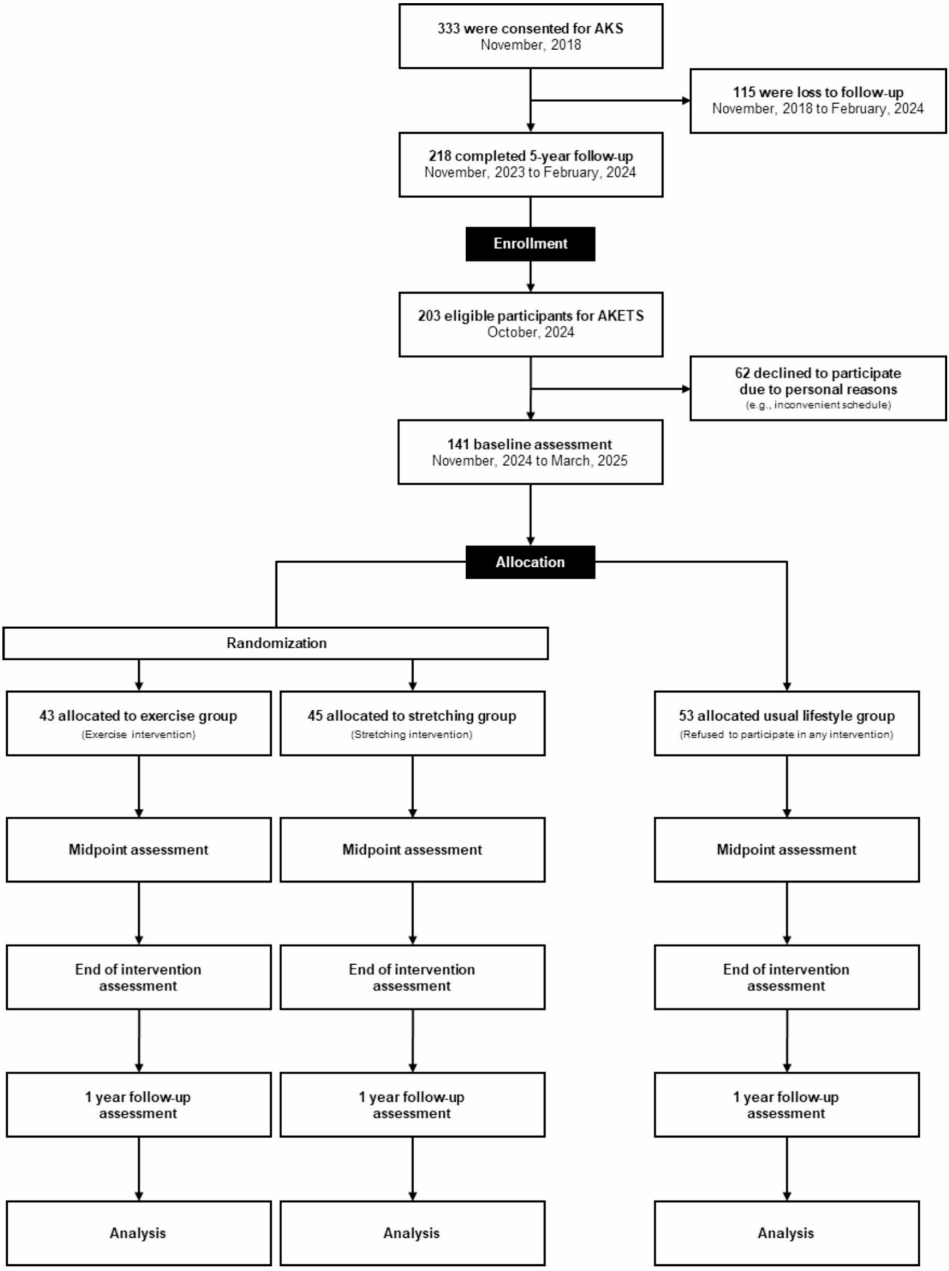
Study flow diagram. Participants will be randomly assigned to either an exercise or stretching intervention program. AKS, Aging Kidney Study; AKETS, Aging Kidney and Exercise Training Study

Participants are to be classified into one of the following three groups: (1) exercise intervention group, (2) stretching intervention (active control) group, and (3) usual lifestyle (time control) group (Fig. 2). Participants voluntarily choose to enroll in either the usual lifestyle group or one of two intervention groups (exercise or stretching). Assignment to the usual lifestyle or intervention group is determined based on participant preference and collected via a questionnaire administered after obtaining written informed consent. Only those who opt for the intervention arm are subsequently randomized into one of the two groups (i.e., exercise or stretching group). Randomization within the intervention arm is stratified by age, sex, and GFR stage and performed using a computer–generated randomization sequence following the completion of baseline assessments. The allocation ratio between the two intervention groups is 1:1. The random allocation sequence was generated by a study statistician (MG). Allocation of participants was conducted by an independent study investigator (YN) who had no direct contact with participants. Personnel involved in participant enrollment (KK) did not have access to the random allocation sequence at any stage, ensuring adequate allocation concealment. The investigators responsible for evaluating the primary outcome are independent of the study team and remain blinded to group allocation throughout the study period. However, due to the inherent characteristics of the interventions (structured exercise training versus stretching) and the need to describe the intervention procedures in the informed consent documents and study protocol, blinding participants to group allocation was not feasible. To mitigate the risk of performance bias, we standardized the frequency, duration, and monitoring procedures of both intervention programs.

### Intervention

#### Exercise intervention group

Participants assigned to the exercise training group participate in an individualized, progressive, multicomponent training program based on the official exercise guidelines of the Japanese Ministry of Health, Labour, and Welfare (https://www.mhlw.go.jp/content/000656460.pdf). The program includes aerobic, resistance, and balance training and is conducted either under supervision at the Medical Fitness Center Phoenix (Tsukuba, Ibaraki, Japan) or unsupervised at home. The participants are instructed to exercise at least three times per week for approximately 60 min per session, with at least one session per week performed under supervision. In addition to each exercise session, warm–up and cool–down activities for approximately 15 min are included. During supervised aerobic sessions, the participants perform 20 min activities using a treadmill or cycle ergometer at an intensity of 50–70% of their age–predicted maximum heart rate calculated using the Tanaka formula (i.e., 208 − 0.7 × age) [44], corresponding to a perceived exertion of “somewhat hard.” Supervised resistance training consists of five machine–based exercises—namely, chest press, seated rowing, leg press, leg curl, and leg extension. Each exercise is performed in 2–3 sets of 10–12 repetitions at 30–70% of the one–repetition maximum (1–RM). The exercise dose (i.e., load, repetitions, and sets) is progressively adjusted based on the participant’s adaptation. The exercise load for months 0–6 is determined using baseline 1–RM, whereas the exercise load for months 7–12 is based on reassessment at 6 months. In unsupervised sessions, participants are encouraged to engage in aerobic activities such as walking, bodyweight resistance exercises (e.g., squats and push–ups), and balance exercises (e.g., front lunges and heel raises). The heart rate is monitored and recorded in real time during all sessions using a wrist–worn device (Xiaomi Smart Band 9, Xiaomi Japan, Tokyo, Japan). To monitor adherence, the participants are required to complete a daily exercise training log documenting their pre–exercise blood pressure, subjective condition, exercise type, duration, and heart rate. Exercise training logs are collected monthly at the Medical Fitness Center Phoenix (Tsukuba, Ibaraki, Japan) and reviewed to assess compliance with the prescribed program. When necessary, follow–up support is provided via telephone or in–person meetings to encourage sustained engagement.

#### Stretching intervention group (Active control group)

To maintain participant motivation and ensure ethical requirements, an active control group is established and provided with a structured stretching intervention program. This program consists of 20 static stretching exercises targeting major muscle groups in the upper body, trunk, and lower body and is designed to minimize cardiovascular load by minimizing heart rate elevation. Following randomization, the participants attend an orientation session in which a certified instructor explains each stretch movement and the procedures for the logging activity. The stretching program is designed to be performed for 10–20 min per session. During the first six months, both the number of stretching exercises and the total session duration are progressively increased. The participants are instructed to perform stretching exercises at home at least three times per week. To monitor adherence, completed stretching logs are collected monthly and reviewed by study personnel.

#### Usual lifestyle group (Time control group)

Within the AKS cohort, a group of participants who are followed up without receiving any intervention is designated as the usual lifestyle (i.e., time control) group for this study. The participants who opt for the time control group are instructed to maintain their usual lifestyle habits and encouraged to participate in annual follow–up assessments.

### Measurements

This study covers four assessment points: baseline, midpoint (6 months), end of intervention (12 months), and follow–up (24 months). Except for magnetic resonance imaging (MRI) and maximal dynamic strength measurements, all assessments are performed at the University of Tsukuba. MRI scans and maximal dynamic strength measurements are scheduled to be performed at the National Institute of Advanced Industrial Science and Technology and Medical Fitness Center Phoenix. After laboratory assessments, the participants also undergo triaxial accelerometer measurements to assess their daily sedentary behavior and physical activity.

### Primary and secondary outcome measures

The primary outcome of this study is the annualized rate of change in eGFR (i.e., eGFR slope), expressed as mL/min/1.73 m² per year. Based on findings from a recent meta–analysis of RCTs, the eGFR slope is recognized as a reliable surrogate endpoint for tracking CKD development [45, 46]. Given that serum cystatin C levels are less influenced by physical activity and muscle mass than serum creatinine levels [47], serum cystatin C levels are used to calculate the eGFR and evaluate changes in kidney function. The eGFR slope is determined using a linear mixed–effects model with random intercepts [48].

The secondary outcomes include alterations in renal hemodynamics and oxygenation assessed using Doppler ultrasound imaging and MRI, glomerular and tubular damage biomarkers, cardiovascular function indices, such as arterial stiffness and endothelial function, blood– and urine–based markers of mineral and bone metabolism, body composition and bone density, muscle strength, cardiorespiratory fitness, physical performance, habitual dietary intake, sedentary behavior, physical activity, sleep quality, and mental health. The detailed measurement methods for the secondary outcomes are provided in the Supplementary Material.

### Assessment of adverse events and adherence

Adverse events will be monitored continuously throughout the study period via monthly participant check–ins, standardized questionnaires, and review of training logs. All reported adverse events will be categorized systematically, with serious adverse events being promptly reviewed by independent clinical advisors. Serious adverse events will be classified if they result in death, are immediately life–threatening, require inpatient hospitalization, or lead to persistent or significant disability/incapacity. Affected participants are free to discontinue the trial or continue with the intervention. Adherence to the intervention, including the frequency and intensity of exercise and stretching (both supervised and home–based), throughout the 12–month intervention period will be assessed using a self–recorded exercise and stretching diary. If a participant drops out of the intervention program, the reasons will be recorded.

### Sample size calculation

The sample size calculation for this clinical trial is based on a previous study that explored the impact of a 1–year structured rehabilitation program on kidney function decline among patients with CKD, using a small sample from a TwiCs study design [49]. According to this previous study, the annual slope of eGFR (mL/min/1.73 m²/year) was approximately + 2 in the intervention group and − 3 in the control group, indicating a between–group difference of approximately 5 units per year. Assuming a standard deviation of 8, a two–sided significance level of 0.05, and a statistical power of 0.80, a total of 84 participants would be required based on a two–sample t–test.

### Statistical analysis

All analyses will be conducted based on the intention–to–treat (ITT) principle. Statistical significance is set at *P* < 0.05. Data are expressed as mean ± standard deviations, median [interquartile range], or frequency counts (%), as appreciate. Baseline group characteristics will be compared using one–way analysis of variance or the Kruskal–Wallis test for continuous variables and the chi–square test or Fisher’s exact test for categorical variables. The eGFR slope for the 6–year pre–intervention, 1–year during–intervention, and 1–year post–intervention phases will be calculated. For calculating the eGFR slope, the repeated measurements of eGFR will be analyzed using a linear mixed model (LMM) including continuous time as a fixed effect and individual participant as a random effect (henceforth, Model 1). In addition, the slope will be estimated using a single linear regression of eGFR on time by each participant (called as Model 2) as a supplementary analysis.

For a primary analysis, the eGFR slope will be compared using the LMM including the group (exercise, stretching, and usual lifestyle), time, their interaction term, and the baseline eGFR as fixed effects and individual participant as a random effect. The eGFR slopes calculated by Models 1 and 2 will also be compared between groups using analysis of covariance, including the group as a factor and the eGFR slope of the 6–year pre–intervention phase as a covariate. The eGFR slope for the 6–year pre–intervention, 1–year during–intervention, and 1–year post–intervention phases will be performed using paired t–tests for within–group comparison.

Secondary outcomes will be analyzed using the generalized estimating equations (GEE) method, including group, categorical time point (baseline, 6 months, and 1 year), and their interaction term as factors. In each model, an unstructured (UN) correlation matrix will be used to account for within–individual correlations across time points. If the UN matrix leads to a convergence problem for numerical optimization, a simpler correlation structure will be used. The results of the GEE analysis will be presented as the marginal means with 95% confidence intervals. Pearson product–moment correlation analysis and repeated–measure correlation analysis [50] will be conducted to examine the associations between individual changes in each outcome.

As a sensitivity analysis, multiple imputations will be applied to the missing outcome data to assess the robustness of the findings from the above analyses. In addition, pre–specified subgroup analyses based on baseline characteristics will be performed to further explore potential effect modification. Specifically, subgroup analyses will be conducted according to baseline kidney function (eGFR < 60 mL/min/1.73 m²), glycemic control status (hemoglobin A1c ≥ 6.5%), hypertension status (systolic blood pressure ≥ 140 mmHg and/or diastolic blood pressure ≥ 90 mmHg), and obesity/overweight (body mass index ≥ 25 kg/m²).

## Results

The demographic and clinical characteristics at the time of allocation of participants who completed the baseline assessment are presented in Table 1. At baseline, the exercise, stretching, and usual lifestyle groups were similar with respect to age, proportion of women, height and weight, body mass index, eGFR, blood biochemistry, blood pressure, and heart rate.

**Table 1:** Baseline characteristics of exercise, stretching, and usual lifestyle groups

	Exercise (*n* = 43)	Stretching (*n* = 45)	Usual lifestyle (*n* = 53)
Age, years	68±9	67±10	69±9
Women, n (%)	32 (74)	32 (71)	37 (70)
Height, cm	157±6	160±10	158±8
Weight, kg	56.3±12.4	57.6±13.6	53.5±10.4
Body mass index, kg/m^2^	22.6±4.1	22.4±4.1	21.2±2.9
Serum cystatin C, mg/dL	0.84±0.19	0.85±0.21	0.92±0.35
eGFR_cys_, mL/min/1.73m^2^	85±19	86±19	81±23
Total cholesterol, mg/dL	220±39	220±37	227±44
HDL cholesterol, mg/dL	76±20	76±19	78±18
LDL cholesterol, mg/dL	122±30	117±27	126±36
Triglyceride, mg/dL	83±35	100±54	84±26
Fasting blood glucose, mg/dL	84±14	90±16	91±16
Hemoglobin A1c, %	5.44±0.43	5.53±0.53	5.48±0.45
Systolic blood pressure, mmHg	125±16	122±15	122±16
Diastolic blood pressure, mmHg	73±8	72±9	72±11
Pulse pressure, mmHg	52±11	50±9	50±9
Heart rate, bpm	60±7	60±9	62±9

Values are presented as the means ± standard deviation or frequency counts (percentage)

eGFR_cys_, estimated glomerular filtration rate calculated based on serum cystatin C levels; HDL, high–density lipoprotein; LDL, low–density lipoprotein

## Discussion

With the rapid aging of the population, the recent predominant demographics of patients has also shifted towards middle–aged and older adults [3]. In this context, the current cases of CKD are mainly associated with the long–term affliction of lifestyle–related diseases, such as diabetes, hypertension, and arteriosclerosis/atherosclerosis. In addition to these pathophysiological trends, considering the progressive nature of CKD without apparent symptoms [5], there is a growing focus on implementing early preventive strategies to minimize the kidney function decline. Numerous epidemiological pieces of evidence demonstrating the independent association of physical activity, performance, and fitness with development in CKD suggest that addressing physical inactivity through regular exercise practices may play a crucial role in preventive strategies against CKD in middle–aged and older adults [29, 30]. However, there is a significant lack of evidence from interventional studies examining whether regularly performed exercise benefits aging kidney health. This TwiCs study will provide intra– and inter–individual comparisons between sedentary and active lifestyles, incorporating regular physical activity and exercise, offering insights into their impact on kidney health in middle–aged and older adults. We will also investigate the residual effects of exercise training intervention throughout the 1–year observation period, as such findings may help understand the potential of sustained preventive strategies for CKD.

A key difference between the handful of previous interventional studies examining the effects of exercise training on kidney health in individuals with or without CKD and our study is the fact that our study has the long–term observational data spanning six years before intervention initiation. This difference is important for addressing the clinical question of whether exercise can improve deteriorating kidney health, which has worsened over several years owing to aging and long–term lifestyle disruptions. To our knowledge, only one pilot study has been conducted with a similar design comparing changes in kidney function before and after the intervention, suggesting that exercise training can slow the progressive deterioration in kidney function in patients with CKD [49]. However, this pilot study had a small sample size (*n* = 20) and short pre–intervention follow–up period (one year) and did not conduct follow–up measurements after the intervention. Therefore, we aimed to address these limitations by adopting a better design (i.e., TwiCs) to clarify whether exercise improves, does not affect, or deteriorates kidney health.

Emerging evidence suggests that the deterioration in renal hemodynamics [12] and mineral metabolism [13] observed in early middle age may contribute to the rapid kidney function decline in later life. Based on these findings, preventive measures to minimize the deterioration of renal hemodynamics and mineral metabolism may lead to improved long–term renal outcomes. However, the long–term effects of preventive interventions on renal hemodynamics and mineral metabolism remain unclear. In this study, as a secondary outcome, we comprehensively investigated the effects of long–term structured exercise training on renal hemodynamics and mineral metabolism in middle–aged and older adults. This will allow us to provide new insights into the underlying mechanisms through which exercise training contributes to the suppression of kidney function decline.

This study has several limitations that should be considered when interpreting our findings. First, due to the voluntary selection of participants into either the intervention or usual lifestyle groups before randomization, there may be potential selection bias; participants opting into intervention groups could systematically differ in baseline health status compared to those choosing the usual lifestyle group. However, as shown in Table 1, the baseline characteristics of exercise, stretching, and usual lifestyle groups were generally comparable, with no substantial differences observed among the three groups. The second concern is the risk of participant dropout. The previous study has shown a completion rate of 80% [49], but this may be optimistic. The extent of dropout could affect the generalizability of the results. To address this concern, we plan to conduct sensitivity analyses to examine the robustness of our findings. Third, the risk of type I error is increased due to the presence of multiple secondary endpoints because the multiplicity of test for the endpoints will not be controlled. Therefore, results from the secondary analyses will be interpreted cautiously and viewed primarily as hypothesis–generating rather than confirmatory. Fourth, in participants with early diabetes or pre–diabetes, transient glomerular hyperfiltration could temporarily elevate eGFR, thereby masking initial declines in kidney function. To mitigate this issue, subgroup analyses stratified by baseline glycemic control status using hemoglobin A1c thresholds (e.g., ≥ 6.5%) will be conducted. Finally, it is important to note that our participants have relatively preserved kidney function compared to populations studied in previous CKD trial [49]; hence, the magnitude of exercise–induced benefits observed in prior CKD–specific studies might not directly generalize to our study population.

In summary, this study is the first long–term RCT in the field of sports nephrology to employ the TwiCs study design. In the context of an ultra–aged society in which the prevalence of CKD is expected to continue to rise, our study provides crucial clinical and physiological insights into the role of regularly performed exercise as a primary prevention for CKD in middle–aged and older adults.

## Supplementary Information

Below is the link to the electronic supplementary material.

Supplementary Material 1

## Data Availability

No datasets were generated or analysed during the current study.
