# Supplementary material for: The Aging Kidney and Exercise Training Study: protocol for a randomized controlled trial within cohorts (TwiCs) study
Source: BMC Nephrol. 2025 Sep 26;26:533. doi: 10.1186/s12882-025-04471-y (PMC12465243; doi:10.1186/s12882-025-04471-y)
Supplement: Supplementary file 1 — Supplementary Material 1 [file 12882_2025_4471_MOESM1_ESM.docx]

**SUPPLEMENTAL METHODS**

**Renal Hemodynamics and Oxygenation**

*Renal Duplex Ultrasonography*

The blood flow profiles in the intrarenal segmental artery are recorded via an ultrasonic diagnostic imaging system with a 1.5–6.0 MHz curved–array convex probe (Vivid E95, General Electric Medical Systems, Milwaukee, WI, USA). During data collection, the participants are instructed to perform mid–exhalation non–Valsalva breath–holds for no longer than 10 sec to minimize the effects of respiration in moving the intrarenal segmental artery [[1](#_ENREF_1)]. Blood flow velocity (BFV) is measured in at least three clips of multiple cardiac cycles from the middle portion of the same intrarenal segmental artery in the right kidney using the coronal approach, with the participants in the left lateral recumbent position. The waveform envelope by time–averaged maximum BFV is used to calculate the mean segmental artery BFV because the sample volume cannot be accurately fitted to the segmental artery diameter [[2](#_ENREF_2)]. Renal vascular resistance is calculated as mean arterial pressure (MAP) divided by mean segmental artery BFV, whereas renal vascular conductance is calculated as mean segmental artery BFV divided by MAP. This methodological approach has been previously described in detail as a Doppler ultrasound–derived measure of intrarenal segmental artery hemodynamics [[3](#_ENREF_3)]. Moreover, the renal pulsatility index and resistive index are calculated using peak systolic and end–diastolic BFVs, which are recorded from the envelope of the renal BFV waveform [[4](#_ENREF_4)].

*Renal* *MRI*

In addition to renal duplex ultrasonography, renal MRI data are collected using a 3T scanner (Ingenia; Philips Medical Systems, Eindhoven, Netherlands) with a 32–channel body coil (dStream Torso) for radiofrequency signal reception. Dixon and blood oxygenation level–dependent (BOLD) MRI sequences are used for renal structure and oxygenation characterization, respectively [[5](#_ENREF_5), [6](#_ENREF_6)].

**Cardiovascular Measurements**

*Blood Pressure and Heart Rate*

Brachial blood pressure and heart rate are measured using a semi–automated vascular testing device with an electrocardiogram, phonocardiogram, and oscillometric extremity cuffs (form PWV/ABI: Model BP203RPEII; Colin Medical Technology, Aichi, Japan).

*Arterial Stiffness*

Brachial–ankle pulse wave velocity, an index of systemic arterial stiffness, is measured using a standard procedure with a semiautomated vascular testing device (Form PWV/ABI: Model BP203RPEII, Colin Medical Technology Corp., Aichi, Japan). In addition to systemic arterial stiffness, central (i.e., carotid) arterial stiffness is assessed using an ultrasonic diagnostic imaging system with an 8.0 MHz linear probe (Vivid E95, General Electric Medical Systems, Milwaukee, WI, USA). Specifically, longitudinal images of the right common carotid artery (1–2 cm proximal to the carotid bulb) are obtained across ≥10 heartbeats. The carotid diameter is measured as the distance between the intima–to–intima layers of the carotid wall. The left carotid arterial pressure waveform is recorded using an applanation tonometry sensor (TU–100; Colin Medical Technology, Aichi, Japan) and calibrated to the brachial mean and diastolic blood pressures. Carotid artery compliance and distensibility are estimated based on intrabeat changes in carotid diameter and blood pressure, as previously described [[7-9](#_ENREF_7)].

*Endothelial Function*

Brachial artery flow–mediated dilation (FMD), the current gold–standard measure of endothelial function, will be measured using a stereotactic probe–holding device equipped with an edge–tracking system for 2D imaging and a pulsed Doppler flow velocimeter for automatic measurement (UNEXEF; UNEX Corp., Aichi, Japan) [[10](#_ENREF_10)]. The FMD values are calculated using the following formula: FMD (%) = ([maximal brachial artery diameter − baseline brachial artery diameter] / baseline brachial artery diameter) × 100 [[10](#_ENREF_10)].

*Pulse Wave Analysis*

Applanation tonometry (TU–100; Colin Medical Technology Corp., Aichi, Japan) is used to record the beat–to–beat carotid pressure waveform, which is converted to an aortic pressure waveform using a validated general transfer function (SphygmoCor version 8.0; AtCor Medical, Sydney, Australia), as described in a previous study [[11](#_ENREF_11)]. The converted aortic blood pressure waveform is calibrated using the MAP and brachial diastolic blood pressure to determine several aortic hemodynamic parameters, including aortic systolic and pulse pressures.

**Blood and Urine Biochemistry**

Blood samples are obtained from the antecubital vein in the morning after an overnight fast. The collected blood samples are centrifuged at 4°C for 15 min at 3,000 rpm using a versatile refrigerated centrifuge (AX–320, TOMY SEIKO Co, Ltd., Tokyo, Japan) and subsequently stored for analysis. Blood parameters (high–density lipoprotein cholesterol, low–density lipoprotein cholesterol, triglycerides, fasting blood glucose, hemoglobin A1c, insulin, creatinine, and cystatin C) are analyzed using validated methods at Tsukuba i–Laboratory LLP (Tsukuba, Ibaraki, Japan). eGFR is calculated using the Japanese eGFR equations based on standardized serum creatinine or cystatin C levels [[12](#_ENREF_12), [13](#_ENREF_13)]. In addition, mineral metabolism markers (e.g., phosphate, calcium, calciprotein particles, fibroblast growth factor 23, 1,25–dihydroxyvitamin D, and intact parathyroid hormone) and bone metabolism markers (e.g., procollagen type 1 N–terminal propeptide, bone–specific alkaline phosphatase, and tartrate–resistant acid phosphatase isoform 5b) are assessed using certified methods.

On the same morning as blood collection, spot urine samples will also be obtained to measure urinary concentrations of creatinine, phosphate, calcium, albumin, liver–type fatty acid–binding protein, β2–microglobulin, and N–acetyl–β–D–glucosaminidase. To account for urine dilution, selected urinary biomarkers are normalized to urinary creatinine concentrations.

**Body Composition and Bone Density**

Body composition is assessed using a stadiometer (AD–6227R; A&D Inc., Tokyo, Japan) and bioelectrical impedance analyzer (InBody 770; InBody Japan Inc., Tokyo, Japan). The InBody 770 device employs a validated direct segmental multi–frequency bioelectrical impedance analysis method to estimate lean mass in both upper limbs [[14](#_ENREF_14)]. Waist circumference is measured in the standing position, whereas lower leg circumference is measured twice in the seated position using a non–elastic measuring tape; the average of the two measurements are used for analysis. Bone density is quantified using a quantitative ultrasound (QUS) device (AOS–100SA; Hitachi–Aloka Medical, Ltd., Tokyo, Japan), which measures the speed of sound and broadband ultrasound attenuation through the right calcaneus [[15](#_ENREF_15), [16](#_ENREF_16)]. Each measurement is performed twice, with the average value being used for analysis. QUS is a non–invasive method for evaluating the risk of osteoporotic fractures and serves as a complementary or alternative tool to dual–energy X–ray absorptiometry in clinical and research settings [[17](#_ENREF_17)].

**Muscle Strength**

*Handgrip Strength*

Handgrip strength will be measured using a Smedley–type dynamometer (GRIP–D, T.K.K.5401; Takei Scientific Instruments Co., Ltd., Niigata, Japan) [[18](#_ENREF_18)]. Each participant will perform two trials with each hand, alternating sides. The participants are instructed to stand in a natural position and squeeze the dynamometer. The maximum value from each hand is determined, with the average of the two highest values (one from each hand) being used for analysis.

*Knee Extensor Strength*

Isometric knee extensor strength is assessed in a seated position with approximately 90° flexion at both the hip and knee joints, using a handheld dynamometer (μTas F–1; ANIMA Corp., Tokyo, Japan), following previously established procedures [[18](#_ENREF_18)]. Participants are instructed to gradually exert maximal force and maintain full knee extension for approximately 3 sec. Peak force during this period is recorded. Each leg is tested twice, with the average of the highest values for each leg being used for analysis. As isometric knee extensor strength is highly influenced by body mass, the values are normalized by body weight.

*Maximal Dynamic Strength*

Maximal dynamic strength (i.e., 1−RM) is assessed for the following exercises using dedicated resistance training machines: leg press, leg extension, leg curl, seated rowing, and chest press. Following an adequate warm–up and familiarization phase, the load is progressively increased so that the participant reaches the maximal load that can be determined within six repetitions. This standardized protocol has been shown to provide highly reliable results while minimizing potential learning effects, particularly among untrained individuals [[19](#_ENREF_19)].

**Cardiorespiratory Fitness**

Cardiorespiratory fitness is objectively assessed using a submaximal exercise test conducted using a cycle ergometer (V77i modified version; Seno Corp., Chiba, Japan). This exercise test consists of a simple 5–6 min incremental load protocol designed to estimate cardiorespiratory fitness. The validity and reliability of this protocol have been reported in previous studies [[20](#_ENREF_20)]. Specifically, maximal oxygen uptake will be estimated based on exercise workload and heart rate during the test using the Åstrand–Rhyming nomogram and correction factors developed by Åstrand [[21](#_ENREF_21), [22](#_ENREF_22)]. The estimated maximal oxygen uptake is used as an index of cardiorespiratory fitness in this study.

**Physical Performance**

*Sit*–*and*–*Reach Test*

Trunk flexibility is assessed using the sit–and–reach test with a digital flexibility–testing device (T.K.K.5412; Takei Scientific Instruments Co., Ltd., Niigata, Japan). The participants are instructed to sit on the floor with their legs fully extended, maintaining contact between the wall and their hips, back, and occiputs, with their arms extended forward and their elbows straight. From this position, they are instructed to slowly reach forward without bending their elbows, and the farthest reach point will be recorded [[23](#_ENREF_23)]. The test is performed twice, and the best value will be used for analysis.

*Five*–*Repetition Sit*–*to*–*Stand*

This test measures the time required to stand up from a 40–cm–high chair and sit back down five times as quickly as possible, without using the hands, while fully extending the knees and hips at each stand [[24](#_ENREF_24)]. Participants are instructed to cross their arms in front of their chest, fully stand upright, and make firm contact with the seat upon sitting. The total time required to complete five repetitions are recorded for analysis.

**Habitual Dietary Intake**

Dietary assessments are conducted using the Brief Diet History Questionnaire (BDHQ). The BDHQ was developed to assess the habitual dietary intake of Japanese adults and includes 73 questions comprising 58 fixed–portion food frequency items and 15 diet history items [[25](#_ENREF_25), [26](#_ENREF_26)]. The validity of the BDHQ was confirmed through comparison with the 16–day dietary records of Japanese adults [[25](#_ENREF_25), [26](#_ENREF_26)].

**Sedentary Behavior and Physical Activity**

Sedentary behavior and physical activity are objectively assessed using a triaxial accelerometer (Active–style Pro HJA–750C; Omron Healthcare Co. Ltd., Kyoto, Japan). This device captures acceleration signals along three axes (anteroposterior, mediolateral, and vertical) and estimates the activity intensity in metabolic equivalents (METs) using a validated built–in algorithm, as demonstrated in previous studies [[27](#_ENREF_27)]. The participants are instructed to wear the device on their left hip for 14 consecutive days, except during bathing or water–based activities, and to record the time of device attachment and removal each day. To ensure data validity, the participants are required to wear the accelerometer for at least 10 hours per day on a minimum of three days, including at least one weekend day [[28](#_ENREF_28)]. Based on the estimated METs, time spent in sedentary behavior (≤1.5 METs), light–intensity physical activity (1.6–2.9 METs), and moderate–to–vigorous physical activity (≥3.0 METs) are calculated. The daily average time spent in each activity category is computed using the following weighted mean: (weekday average × 5 + weekend average × 2) / 7. To minimize behavioral reactivity, the device screen is restricted to display only time.

**Self**–**Reported Sleep Quality, Pain, and Mental Health**

Sleep quality is assessed using the Pittsburgh Sleep Quality Index (PSQI). The Japanese version of the PSQI has been shown to be a reliable and valid instrument for evaluating subjective sleep quality in both clinical and research settings [[29](#_ENREF_29)]. Subjective pain and depressive symptoms are assessed using a standardized pain scale and the Beck Depression Inventory–Second Edition, respectively, to evaluate both the presence and severity of these conditions.

**REFERENCES**

1. Chapman CL, Benati JM, Johnson BD, Vargas NT, Lema PC, Schlader ZJ: **Renal and segmental artery hemodynamics during whole body passive heating and cooling recovery**. *J Appl Physiol (1985)* 2019, **127**(4):974-983.

2. Blanco P: **Volumetric blood flow measurement using Doppler ultrasound: concerns about the technique**. *J Ultrasound* 2015, **18**(2):201-204.

3. Chapman CL, Johnson BD, Hostler D, Lema PC, Schlader ZJ: **Reliability and agreement of human renal and segmental artery hemodynamics measured using Doppler ultrasound**. *J Appl Physiol (1985)* 2020, **128**(3):627-636.

4. Kosaki K, Tarumi T, Sugawara J, Tanahashi K, Kumagai H, Matsui M, Sugaya T, Osuka Y, Tanaka K, Kuro-o M *et al*: **Renal hemodynamics across the adult lifespan: Relevance of flow pulsatility to chronic kidney disease**. *Exp Gerontol* 2021, **152**:111459.

5. Inoue T, Kozawa E, Ishikawa M, Fukaya D, Amano H, Watanabe Y, Tomori K, Kobayashi N, Niitsu M, Okada H: **Comparison of multiparametric magnetic resonance imaging sequences with laboratory parameters for prognosticating renal function in chronic kidney disease**. *Sci Rep* 2021, **11**(1):22129.

6. Inoue T, Kozawa E, Okada H, Inukai K, Watanabe S, Kikuta T, Watanabe Y, Takenaka T, Katayama S, Tanaka J *et al*: **Noninvasive evaluation of kidney hypoxia and fibrosis using magnetic resonance imaging**. *J Am Soc Nephrol* 2011, **22**(8):1429-1434.

7. Mori S, Tarumi T, Kosaki K, Matsui M, Yoshioka M, Sugawara J, Kuro-o M, Saito C, Yamagata K, Maeda S: **Effects of the number of sit-stand maneuver repetitions on baroreflex sensitivity and cardiovascular risk assessments**. *Am J Physiol Regul Integr Comp Physiol* 2022, **322**(5):R400-R410.

8. Maeda S, Sugawara J, Yoshizawa M, Otsuki T, Shimojo N, Jesmin S, Ajisaka R, Miyauchi T, Tanaka H: **Involvement of endothelin-1 in habitual exercise-induced increase in arterial compliance**. *Acta Physiol (Oxf)* 2009, **196**(2):223-229.

9. Van Merode T, Hick PJ, Hoeks AP, Rahn KH, Reneman RS: **Carotid artery wall properties in normotensive and borderline hypertensive subjects of various ages**. *Ultrasound Med Biol* 1988, **14**(7):563-569.

10. Thijssen DH, Black MA, Pyke KE, Padilla J, Atkinson G, Harris RA, Parker B, Widlansky ME, Tschakovsky ME, Green DJ: **Assessment of flow-mediated dilation in humans: a methodological and physiological guideline**. *Am J Physiol Heart Circ Physiol* 2011, **300**(1):H2-12.

11. Kosaki K, Kamijo-Ikemori A, Sugaya T, Tanahashi K, Kumagai H, Sawano Y, Akazawa N, Osuka Y, Tanaka K, Kimura K *et al*: **Urinary liver-type fatty acid-binding protein is associated with subendocardial viability ratio in middle- and older-aged adults**. *Clin Exp Hypertens* 2018, **40**(3):244-250.

12. Horio M, Imai E, Yasuda Y, Watanabe T, Matsuo S, Collaborators Developing the Japanese Equation for Estimated GFR: **GFR estimation using standardized serum cystatin C in Japan**. *Am J Kidney Dis* 2013, **61**(2):197-203.

13. Matsuo S, Imai E, Horio M, Yasuda Y, Tomita K, Nitta K, Yamagata K, Tomino Y, Yokoyama H, Hishida A *et al*: **Revised equations for estimated GFR from serum creatinine in Japan**. *Am J Kidney Dis* 2009, **53**(6):982-992.

14. Ling CH, de Craen AJ, Slagboom PE, Gunn DA, Stokkel MP, Westendorp RG, Maier AB: **Accuracy of direct segmental multi-frequency bioimpedance analysis in the assessment of total body and segmental body composition in middle-aged adult population**. *Clin Nutr* 2011, **30**(5):610-615.

15. Tsuda-Futami E, Hans D, Njeh CF, Fuerst T, Fan B, Li J, He YQ, Genant HK: **An evaluation of a new gel-coupled ultrasound device for the quantitative assessment of bone**. *Br J Radiol* 1999, **72**(859):691-700.

16. Yoshioka M, Kosaki K, Matsui M, Shibata A, Oka K, Kuro-o M, Saito C, Yamagata K, Maeda S: **Replacing sedentary time for physical activity on bone density in patients with chronic kidney disease**. *J Bone Miner Metab* 2021, **39**(6):1091-1100.

17. Njeh CF, Boivin CM, Langton CM: **The role of ultrasound in the assessment of osteoporosis: a review**. *Osteoporos Int* 1997, **7**(1):7-22.

18. Yoshioka M, Kosaki K, Matsui M, Takahashi K, Shibata A, Oka K, Kuro-o M, Saito C, Yamagata K, Maeda S: **Physical Activity, Sedentary Behavior, and Skeletal Muscle Strength in Patients With Chronic Kidney Disease: An Isotemporal Substitution Approach**. *Phys Ther* 2021, **101**(7).

19. Levinger I, Goodman C, Hare DL, Jerums G, Toia D, Selig S: **The reliability of the 1RM strength test for untrained middle-aged individuals**. *J Sci Med Sport* 2009, **12**(2):310-316.

20. Zhai X, Sawada SS, Kurosawa S, Koriyama S, Dimitroff SA, Sato S, Oguma Y, Nakata Y, Maruo K, Miyachi M *et al*: **Cardiorespiratory fitness and body mass index on metabolic syndrome in middle-aged Japanese adults under national health guidance: a cross-sectional study**. *BMC Public Health* 2024, **24**(1):2050.

21. Astrand I: **Aerobic work capacity in men and women with special reference to age**. *Acta Physiol Scand Suppl* 1960, **49**(169):1-92.

22. Astrand PO, Ryhming I: **A nomogram for calculation of aerobic capacity (physical fitness) from pulse rate during sub-maximal work**. *J Appl Physiol* 1954, **7**(2):218-221.

23. Nishitani N, Kosaki K, Matsui M, Sugaya T, Kuro-o M, Saito C, Yamagata K, Maeda S: **Association between trunk flexibility and renal flow pulsatility in middle-aged and older adults**. *Exp Gerontol* 2023, **172**:112060.

24. Bohannon RW, Bubela DJ, Magasi SR, Wang YC, Gershon RC: **Sit-to-stand test: Performance and determinants across the age-span**. *Isokinet Exerc Sci* 2010, **18**(4):235-240.

25. Kobayashi S, Honda S, Murakami K, Sasaki S, Okubo H, Hirota N, Notsu A, Fukui M, Date C: **Both comprehensive and brief self-administered diet history questionnaires satisfactorily rank nutrient intakes in Japanese adults**. *J Epidemiol* 2012, **22**(2):151-159.

26. Kobayashi S, Murakami K, Sasaki S, Okubo H, Hirota N, Notsu A, Fukui M, Date C: **Comparison of relative validity of food group intakes estimated by comprehensive and brief-type self-administered diet history questionnaires against 16 d dietary records in Japanese adults**. *Public Health Nutr* 2011, **14**(7):1200-1211.

27. Ohkawara K, Oshima Y, Hikihara Y, Ishikawa-Takata K, Tabata I, Tanaka S: **Real-time estimation of daily physical activity intensity by a triaxial accelerometer and a gravity-removal classification algorithm**. *Br J Nutr* 2011, **105**(11):1681-1691.

28. Kosaki K, Takahashi K, Matsui M, Yoshioka M, Mori S, Nishitani N, Shibata A, Saito C, Kuro-o M, Yamagata K *et al*: **Sedentary behavior and estimated nephron number in middle-aged and older adults with or without chronic kidney disease**. *Exp Gerontol* 2021, **154**:111531.

29. Doi Y, Minowa M, Uchiyama M, Okawa M, Kim K, Shibui K, Kamei Y: **Psychometric assessment of subjective sleep quality using the Japanese version of the Pittsburgh Sleep Quality Index (PSQI-J) in psychiatric disordered and control subjects**. *Psychiatry Res* 2000, **97**(2-3):165-172.
